# En-bloc lung transplantation: Rare but useful

**DOI:** 10.1016/j.xjtc.2026.102302

**Published:** 2026-03-13

**Authors:** John W. O'Neill, Aly Sherif Hassaballa, Paulo Gregorio, Zane Mazur, Maria Crespo, Christian Bermudez

**Affiliations:** a Division of Cardiac Surgery, Hospital of the University of Pennsylvania, Philadelphia, Pa; b Division of Pulmonary Medicine, Hospital of the University of Pennsylvania, Philadelphia, Pa

**Figure:**
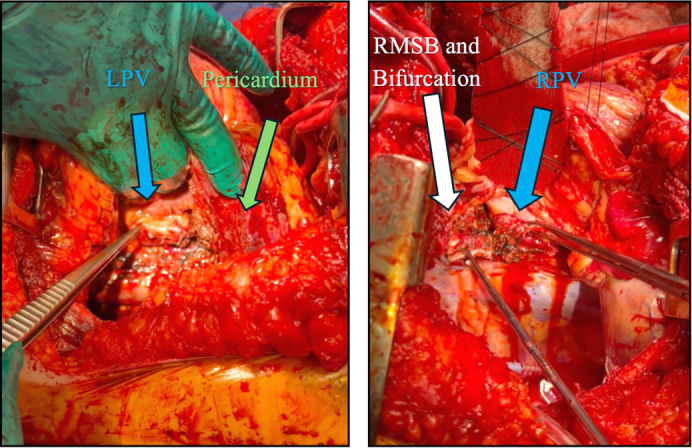
Left pericardial (*left panel*) and right pericardial (*right panel*) windows in EBLT.

**Central Message**

Although uncommon, EBLT serves as an important option for certain patients with central airway pathology.

Lung transplantation is an established therapy for patients with advanced lung disease. Dark and colleagues^1^ first described en-bloc lung transplantation (EBLT) in a canine model in 1986, shortly thereafter in primates, and ultimately Patterson and colleagues^2^ performed the first successful human EBLT in 1988 in a 42-year-old female patient with emphysema. Although now replaced by sequential double-lung transplantation as the result of concerns of tracheal dehiscence secondary to ischemia, EBLT is a viable option for patients with proximal airway disease or when there is otherwise an inability to perform direct bronchial anastomosis.^3^, ^4^, ^5^

Here, we describe the use of EBLT with a tracheal anastomosis and omental flap in a young female patient with progressive bilateral bronchial stenosis and bronchiectasis who had been managed with frequent airway dilations since 5 years of age and had required extracorporeal membrane oxygenation support 5 years before this admission (Videos 1-4). During an attempted dilation at our facility, she developed type 2 respiratory failure and was placed on venovenous extracorporeal membrane oxygenation as a bridge to transplant, ultimately performed 30 days later.

## Surgical Procedure

### Chest Entry, Flap Mobilization, and Bilateral Pneumonectomies

**Figure 1:**
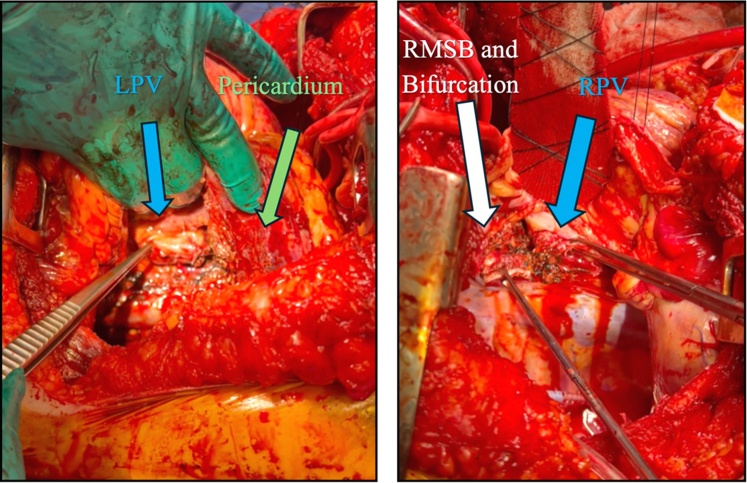
Left pericardial (*left panel*) and right pericardial (*right panel*) windows in en-bloc lung transplantation. *LPV*, Left pulmonary vein; *RMSB*, right mainstem bronchus; *RPV*, right pulmonary.

**Figure E1:**
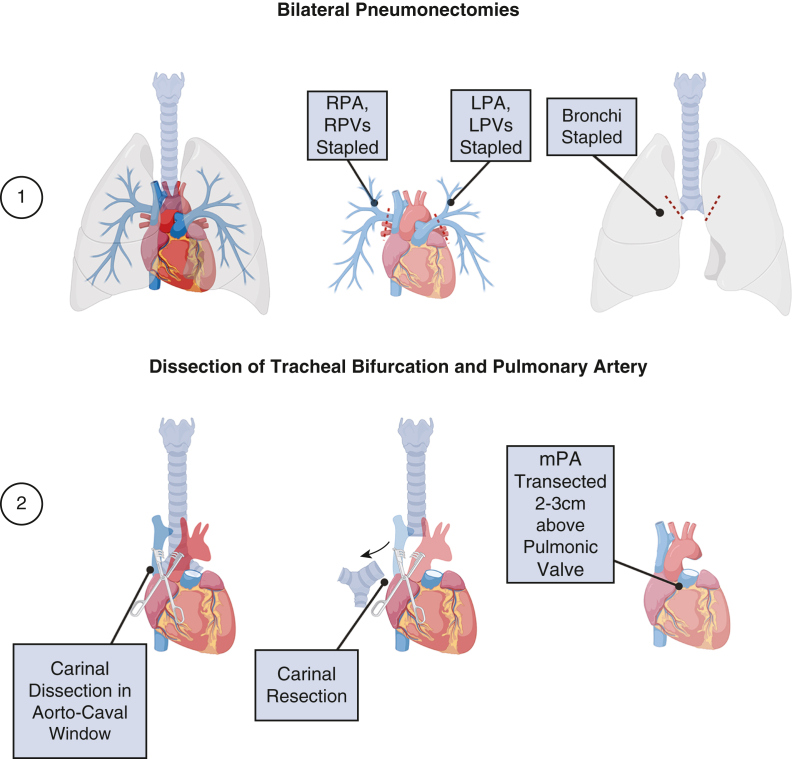
Recipient preparation. #1, bilateral pneumonectomies, and #2, dissection of tracheal bifurcation and pulmonary artery.

After the placement of a single lumen proximal endotracheal tube and a Swan-Ganz catheter (Edwards Lifesciences), the patient was positioned in standard fashion. We performed a bilateral thoracosternotomy through the fifth intercostal space. Encountering substantial adhesive disease, we proceeded cautiously with blunt dissection and cautery to avoid phrenic nerve injury while separating lung tissue and pericardium. An omental flap was mobilized via a small upper abdominal midline incision and tunneled through an anterior diaphragmatic window. The pericardium was opened longitudinally, and standard retraction sutures were placed. To allow for safe hilar dissection, we proceeded with full cardiopulmonary bypass. The existing venous cannulas (right common femoral vein, right internal jugular vein) were converted to serve as dual drainage and the patient was heparinized to obtain an activated clotting time greater than 400 seconds. An ascending aortic cannula was placed via modified Seldinger technique and flows were set >2.2 L/min/m^2^. Beginning with the right hilum, the pulmonary artery (PA) and veins were surrounded individually with umbilical tapes and divided with vascular staplers followed by division of the bronchus with a TA-60 stapler (Figure E1). A right atrial vent was placed as the result of right ventricular (RV) dilation. We completed the left pneumonectomy in a similar fashion. Significant adhesions were noted between the left PA and the pericardium. To avoid risk of phrenic nerve injury with further dissection, we used an additional stapler load to divide the left PA in an intrapericardial fashion, leaving roughly 2 cm of isolated PA adherent to the pericardium. After freeing the pericardial sac and mobilizing the posterior wall of the left atrium with assisted countertension from the caval snare, we created wide left and then right pericardial windows beneath the respective phrenic nerves using blunt dissection and cautery (Figure 1).

### Donor and Recipient Preparation

En-bloc donor lungs were procured with a large left atrial cuff, a long main PA, and a proximal tracheal transection. The donor heart was transplanted at another center. Antegrade and retrograde flushes of Perfadex and intravenous prostaglandin were performed. The recipient's distal trachea was mobilized from the PA and to the right via the posterior planes using blunt dissection. With the recipient's trachea exposed centrally in a posterior pericardial window created between the recipient's aorta and superior vena cava, the recipient's tracheal bifurcation was then resected. Bilateral tracheal retraction sutures were placed to gently assist with anterior mobilization and dissection was limited to minimize ischemia. The recipient's main PA was then divided 2 to 3 cm from the pulmonic valve. Mobilizing the heart superiorly, the donor lungs were placed posterior to the heart, and each lung was tunneled through its respective pericardial window. A self-retaining retractor improved visualization of the space between the aorta and superior vena cava and the tracheal ends were then brought into proximity.

### Anastomoses and Case Conclusion

**Figure E2:**
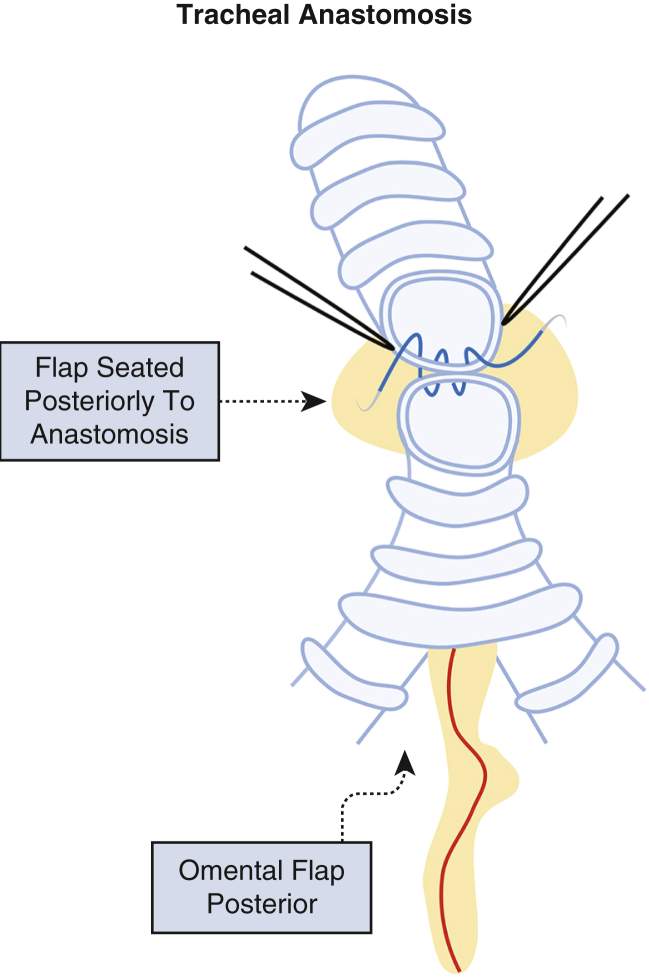
Tracheal anastomosis. *Solid black lines* represent retention sutures.

**Figure E3:**
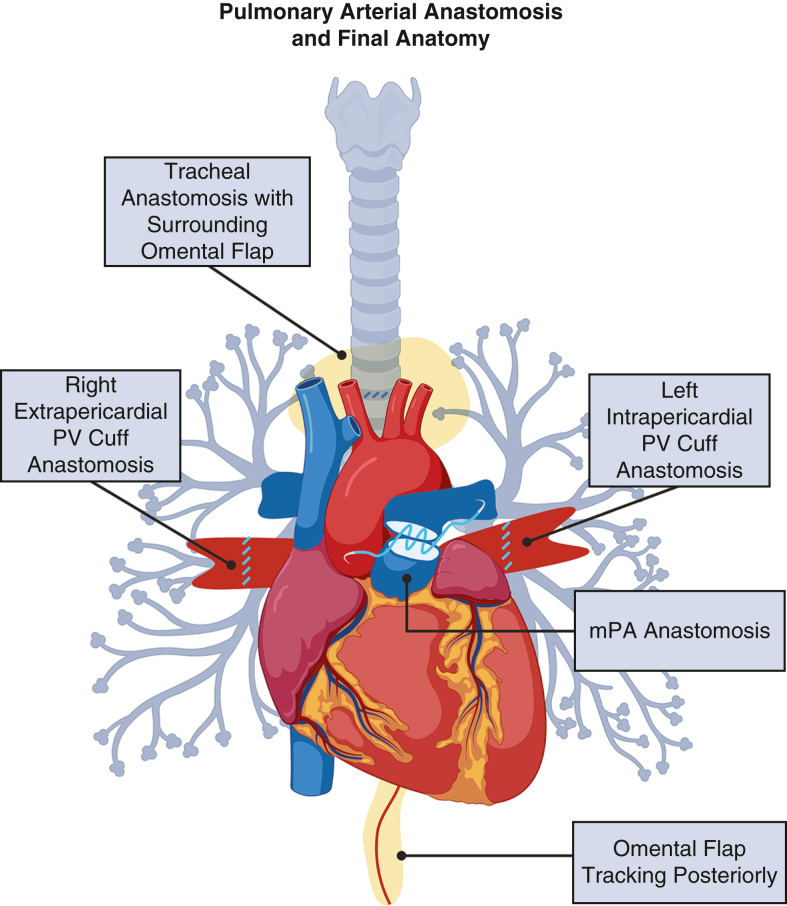
Completion anatomy. Final location of omental flap after tracheal, pulmonary vein cuff, and main pulmonary artery anastomoses.

Once we were satisfied with recipient and donor tracheal length, an end-to-end tracheal anastomosis was performed using running 3-0 PROLENE (Ethicon; Figure E2). The pericardium was then tacked to cover the anterior aspect, and the omental flap was positioned posteriorly around the anastomosis. The extrapericardial right pulmonary vein cuff anastomosis was completed in running fashion with 4-0 PROLENE. The left pulmonary vein cuff anastomosis was performed in an intrapericardial fashion to facilitate exposure. Finally, the recipient and donor main PA were sown with running 5-0 PROLENE in an end-to-end fashion (Figure E3).

**Figure E4:**
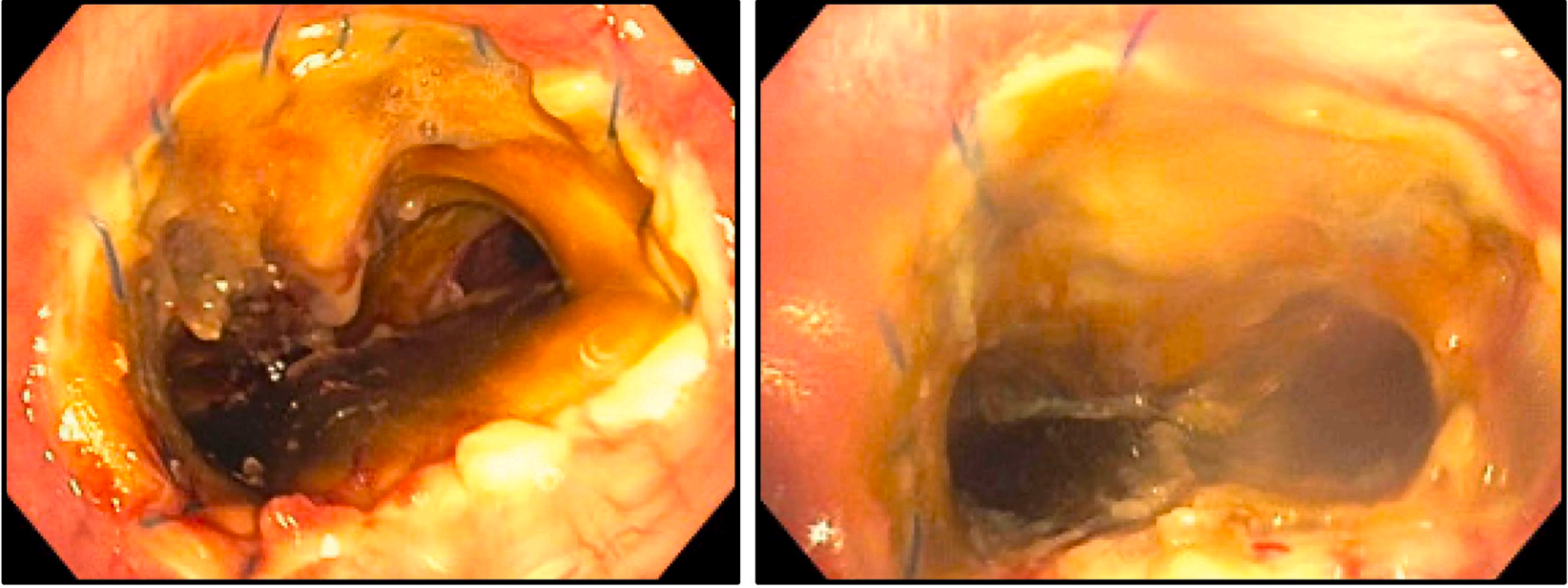
Early bronchoscopy. Shown are 1-month (*left panel*) and 2-month (*right panel*) views of the tracheal bifurcation.

**Figure E5:**
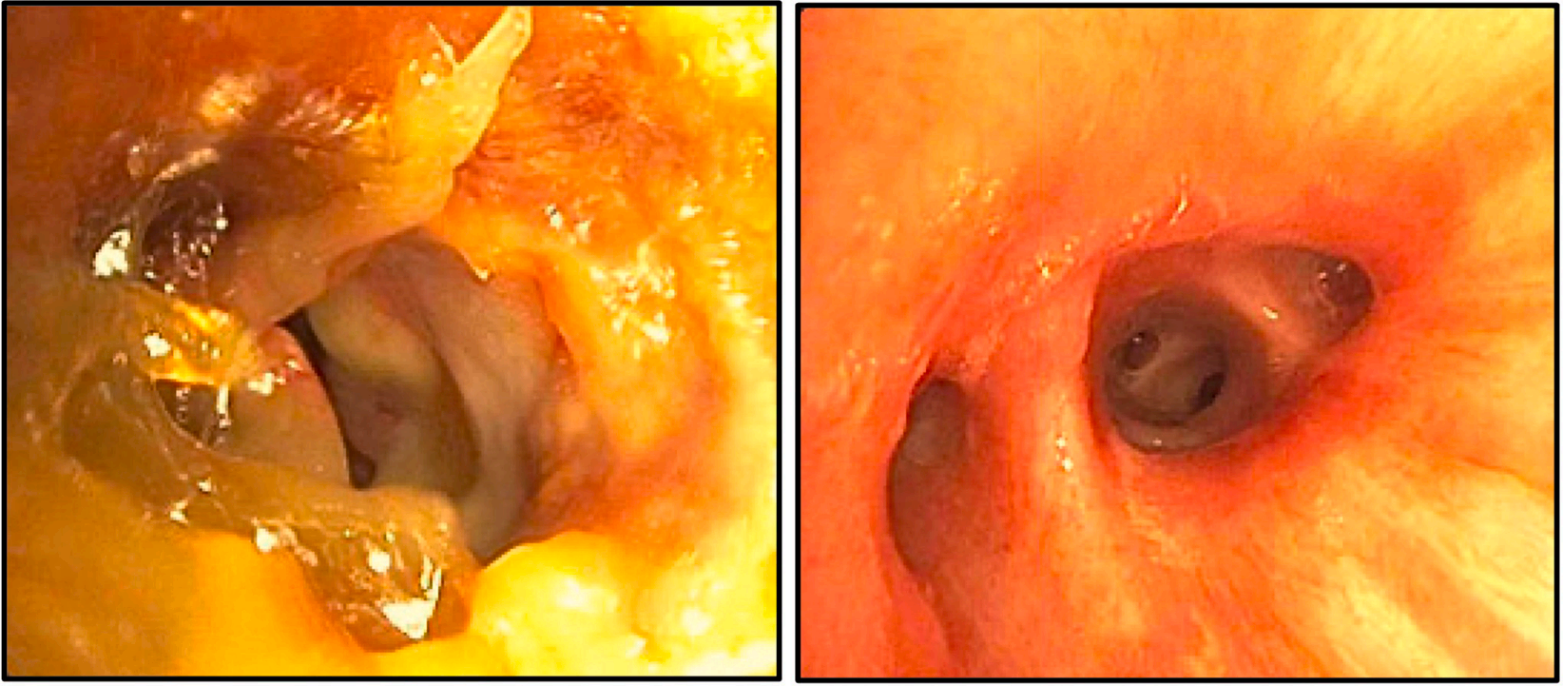
Representative 4-month postoperative bronchoscopy images of the left mainstem bronchus with visible mucus (*left panel*) as well as the distal right mainstem bronchus (*right panel*).

**Figure E6:**
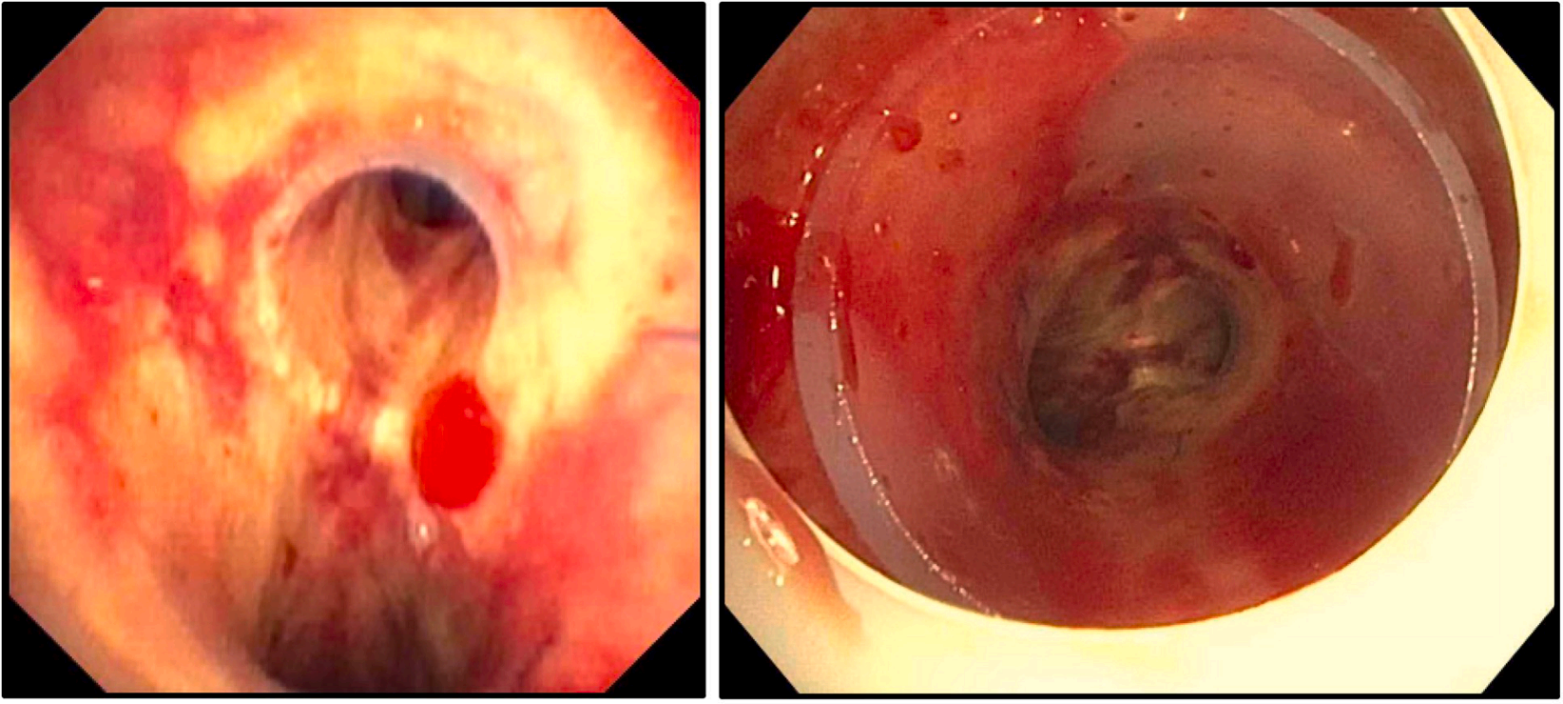
Airway stent. Views at the carina (*left panel*) and trachea (*right panel*) after placement of a custom “Y” stent approximately 4 and a half months from transplantation.

After implantation, an aortic root vent was placed, caval snares were released, and all anastomoses were inspected while mechanical ventilation was initiated. Once satisfied, the right atrial and aortic root vents were removed. Transesophageal echocardiography noted a left ventricular ejection fraction of 55% to 60% with mild to moderate reduction in RV function while on 4 μg/min of epinephrine. We came off bypass after 260 minutes, protamine was administered, and the aortic cannula was removed. Aortic sutures were reinforced with 4-0 PROLENE on pledgets, and venous cannulas were removed. The chest was left open because of the potential for bleeding and to avoid RV compression. Three chest tubes were placed bilaterally and the incision was covered with Esmark (Cardinal Health) and Ioban (3M). Arterial oxygen tension on 60% FiO_2_ was 216 mmHg, and the tracheal anastomosis was patent on bronchoscopy. The patient's chest was closed 3 days after transplant. Bronchoscopy revealed ischemia in the distal donor trachea, which was ultimately managed with serial bronchoscopic debridement and a temporary left bronchial stent. To facilitate this, an open tracheostomy was performed on postoperative day 7. The patient did not experience primary graft dysfunction and was weaned from the ventilator on postoperative day 28. Figures E4 and E5 demonstrate more recent bronchoscopic views. After the placement of a custom “Y” stent for recurrent proximal left bronchial stenosis (Figure E6), the patient discharged with New York Heart Association class I functional status approximately 4 and a half months from transplantation.

## Conflict of Interest Statement

The authors reported no conflicts of interest.

The *Journal* policy requires editors and reviewers to disclose conflicts of interest and to decline handling or reviewing manuscripts for which they may have a conflict of interest. The editors and reviewers of this article have no conflicts of interest.

## Supplementary Data

Video 1 Chest entry. Video available at: https://www.jtcvs.org/article/S2666-2507(26)00109-4/fulltext.

Video 2 Pneumonectomies. Video available at: https://www.jtcvs.org/article/S2666-2507(26)00109-4/fulltext.

Video 3 Implantation. Video available at: https://www.jtcvs.org/article/S2666-2507(26)00109-4/fulltext.

Video 4 Conclusion. Video available at: https://www.jtcvs.org/article/S2666-2507(26)00109-4/fulltext.
