# Supplementary figures and images for: En-bloc lung transplantation: Rare but useful
Source: JTCVS Tech. 2026 Mar 13;37:102302. doi: 10.1016/j.xjtc.2026.102302 (PMC13261281; doi:10.1016/j.xjtc.2026.102302)

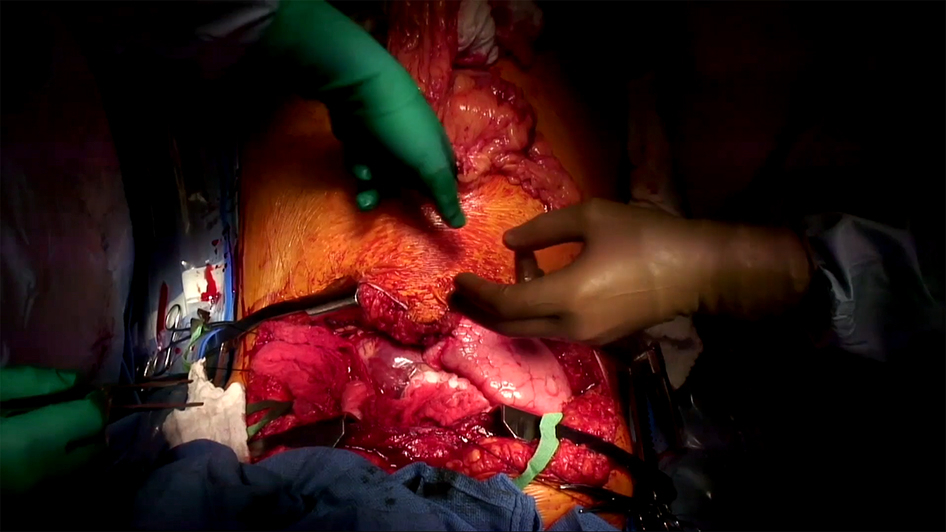

Supplement: Video 1 — Chest entry. Video available at: https://www.jtcvs.org/article/S2666-2507(26)00109-4/fulltext. [file fx2.jpg]

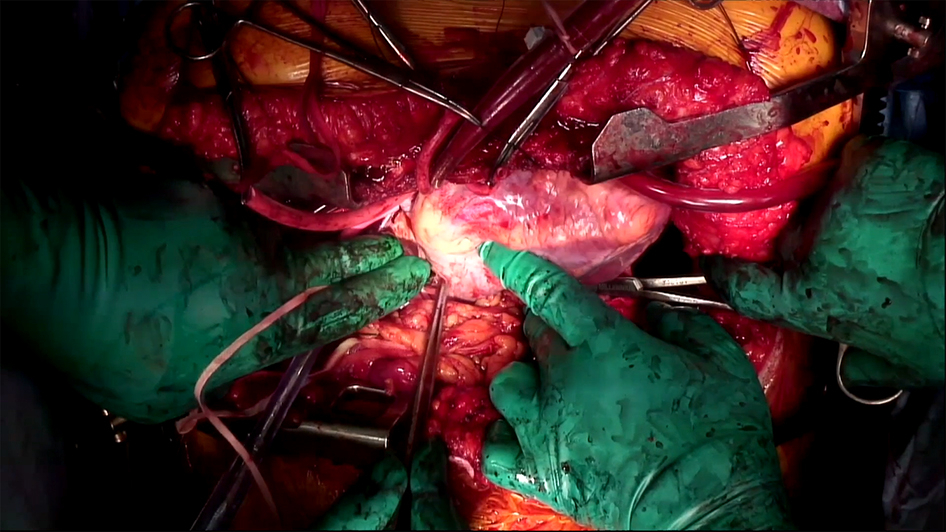

Supplement: Video 2 — Pneumonectomies. Video available at: https://www.jtcvs.org/article/S2666-2507(26)00109-4/fulltext. [file fx3.jpg]

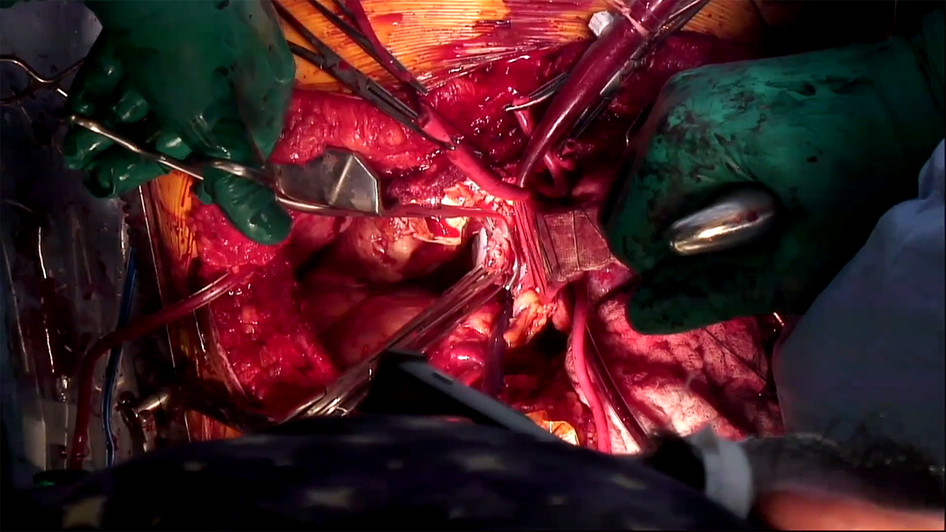

Supplement: Video 3 — Implantation. Video available at: https://www.jtcvs.org/article/S2666-2507(26)00109-4/fulltext. [file fx4.jpg]

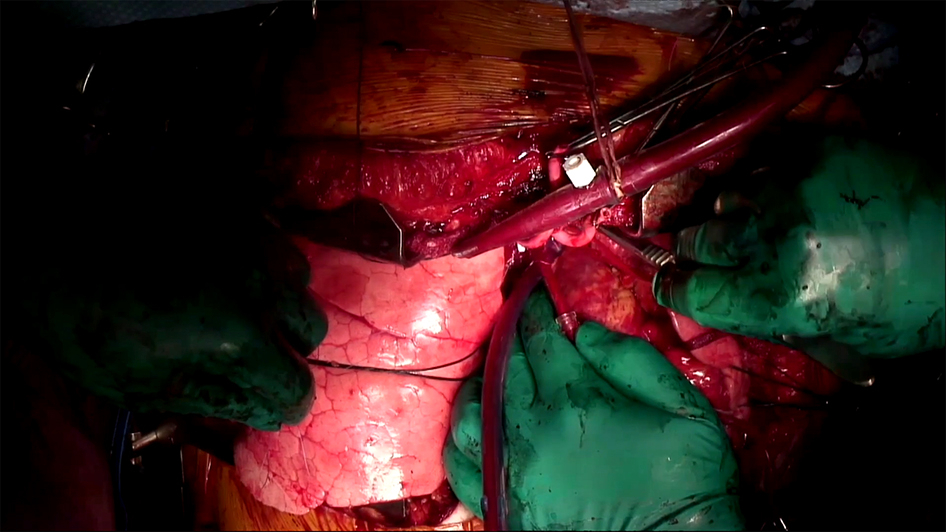

Supplement: Video 4 — Conclusion. Video available at: https://www.jtcvs.org/article/S2666-2507(26)00109-4/fulltext. [file fx5.jpg]
